# Supplementary material for: Performance Evaluation of Machine Learning Algorithms for Sarcopenia Diagnosis in Older Adults
Source: Healthcare (Basel). 2023 Oct 9;11(19):2699. doi: 10.3390/healthcare11192699 (PMC10572141; doi:10.3390/healthcare11192699)
Supplement: Supplementary file 1 [file healthcare-11-02699-s001.zip › healthcare-2610212-supplementary.pdf]

Supplementary Materials

Supplementary Table S1. SARC-F Screen for Sarcopenia (https://www.physio-pedia.com/SARC-F:_A_Simple_Questionnaire_to_Rapidly_Diagnose_Sarcopenia).

SARC-F Screen for Sarcopenia: Component Question Scoring
*Strength: How much difficulty do you have in lifting and carrying 10 pounds?
- None 0
- Some 1
- A lot or unable 2

*Assistance in walking: How much difficulty do you have walking across a room?
- None 0
- Some 1
- A lot, use aids, or unable 2

*Rise from a chair: How much difficulty do you have transferring from a chair or bed?
- None 0
- Some 1
- A lot or unable without help 2

*Climb stairs: How much difficulty do you have climbing a flight of 10 stairs?
- None 0
- Some 1
- A lot or unable 2

*Falls: How many times have you fallen in the past year?
- None 0
- 1ess than 3 falls 1
- 4 or more falls 2

Data suggests that a SARC-F score of ≥4 best predicts the need for further, more comprehensive evaluation.

Supplementary Table S2. Hyperparameters of the Machine Learning Models.

For ALL Models					
**Parameters**	**Values**	**Parameters**	**Values**	**Parameters**	**Values**
**Algorithm name**	XGBoost	**Algorithm name**	Random Forest	**Algorithm name**	Light GBM
**eta**	0.05	**criterion**	gini	**min data in leaf**	3
**gamma**	0.001	**max_features**	11	**max depth**	5
**max_depth**	6	**min_samples_leaf**	30	**num leaves**	62
**max_leaves**	2	**min_samples_split**	10	**num iterations**	1000
**reg_alpha**	0.8333	**ntrees**	1000	**learning rate**	0.1
**reg_lambda**	0.9149			**bagging fraction**	0.8
**subsample**	0.5				
**For Female Models**					
**Parameters**	**Values**	**Parameters**	**Values**	**Parameters**	**Values**
**Algorithm name**	SVM	**Algorithm name**	Random Forest	**Algorithm name**	kNN
**C**	1.206	**criterion**	gini	**metric**	cityblock
**kernel**	rbf	**max_features**	11	**n_neighbors**	2
		**min_samples_leaf**	30	**weights**	distance
		**min_samples_split**	10		
		**ntrees**	1000		

Supplementary Table S3. Average compute time (minutes).

	Time (minutes)
**XGBoost-ALL model**	14.04
**Random Forest-ALL model**	15.57
**LightGBM-ALL model**	13.18
**Support Vector Machine-Female Model**	15.20
**Random Forest-Female model**	14.33
**K Nearest Neighbors-Female Model**	13.49

Supplemantary Table S4. Results of the Training Set

ALL Model*	LightGBM	RF	XGBoost
**Accuracy**	0.939	0.907	0.878
**AUC**	0.984	0.949	0.941
**F1 score**	0.934	0.882	0.869
**Matthews Correlation Coefficient**	0.687	0.555	0.551
**Precision**	0.943	0.906	0.880
**Recall**	0.937	0.914	0.887
**Female Model***	**SVM**	**RF**	**KNN**
**Accuracy**	0.954	0.938	0.931
**AUC**	0.995	0.972	0.966
**F1 score**	0.930	0.946	0. 943
**Matthews Correlation Coefficient**	0.691	0.799	0.711
**Precision**	0.949	0.968	0.935
**Recall**	0.943	0.971	0.873

*** Results of the Training Set**
